# Application of Home-based extended care based on cloud follow-up in postoperative rehabilitation of lower extremity arteriosclerosis obliterans

**DOI:** 10.3389/fmed.2026.1737782

**Published:** 2026-08-19

**Authors:** Yuandan Huang, Yanjun Zhang, Yang Xu, Peng Wang

**Affiliations:** Department of Interventional Radiology, The First Hospital of China Medical University, Shenyang, China

**Keywords:** cloud follow-up, Home-based extended care, lower extremity arteriosclerosis obliterans, quality of life, rehabilitation

## Abstract

**Objective:**

To explore the application effect of Home-based extended care based on cloud follow-up in the postoperative rehabilitation of lower limb arteriosclerosis obliterans and provide a reference for postoperative rehabilitation of lower limb arteriosclerosis obliterans.

**Methods:**

This study employed a prospective cohort study design. 110 patients with lower extremity arteriosclerosis obliterans who underwent interventional treatment were selected and divided into a control group (*n* = 52) and an experimental group (*n* = 58) based on different postoperative management methods. After discharge, the control group received routine management, and the experimental group received Home-based extended care based on cloud follow-up. The differences in recurrence, lower extremity arterial perfusion status, treatment adherence, motor function, and quality of life between the two groups were compared.

**Results:**

Regarding vascular outcomes, the restenosis rate at 12 months post-discharge was 18.97% in the experimental group, which was significantly lower than the 36.54% observed in the control group (*p* < 0.05). Six months after discharge, the adherence of the experimental group with medication, diet control, rehabilitation exercise and regular review was higher than that of the control group (*p* < 0.05). After adjusting for baseline values using analysis of covariance (ANCOVA), the experimental group demonstrated significantly superior brachial-ankle index, Fugl-Meyer Assessment scores, 6MWD, and all VascuQol domains compared with the control group at 12 months post-discharge (*p* < 0.05).

**Conclusion:**

Home-based extended care based on cloud follow-up for the rehabilitation of patients with lower extremity arteriosclerosis obliterans can overcome the temporal and spatial constraints of out-of-hospital care. By enhancing the practicality, specificity, and professionalism of rehabilitation guidance, this approach is associated with superior recovery outcomes, including reduced postoperative restenosis, improved lower limb arterial perfusion, and enhanced treatment adherence, motor function, and quality of life.

## 1 Introduction

Lower extremity arteriosclerosis obliterans is a chronic limb arteriosclerotic disease caused by atherosclerotic lesions (1). With the continuous increase in the incidence of hypertension, diabetes and other diseases in my country and the continuous aggravation of aging, the incidence of lower extremity arteriosclerosis obliterans has increased year by year. Lower extremity arteriosclerosis obliterans will directly affect the blood supply to the lower extremities, causing fatigue, rest pain and intermittent claudication in the affected limbs. In severe cases, it can induce lower extremity ulcers or gangrene, seriously damaging the patient’s lower extremity function and quality of life (2). Interventional surgery is currently the main treatment for lower extremity arteriosclerosis obliterans, but interventional surgery does not completely eliminate the potential factors that induce the disease, and there is still a risk of disease recurrence after surgery (3). After discharge, patients need to adhere to lifelong treatment, take medication, maintain a healthy lifestyle, and have regular follow-up visits to improve prognosis and prevent vascular restenosis (4). Surveys and studies have found that patients with lower extremity arteriosclerosis obliterans have insufficient knowledge of health after surgery, and their compliance with medication and rehabilitation exercises is poor (5). Hospitals should provide patients with health care, rehabilitation exercises, supervision and management and other medical payment services after discharge. Clinical studies have confirmed that strengthening the continuous follow-up and guidance of chronic disease patients after discharge can promote patients to maintain a healthy lifestyle, improve medication and rehabilitation compliance, and enhance patients’ confidence in adhering to treatment through positive and effective psychological guidance (6).

As the coverage of “Internet + medical health” services expands, the advantages of the “Internet +” management model in expanding the supply and efficiency of medical services are gradually emerging. Evidence from multiple studies on Internet-platform-based health management for patients with peritoneal dialysis and coronary heart disease has confirmed its effectiveness in alleviating the shortage of healthcare resources and overcoming the temporal and spatial constraints of conventional follow-up, thereby significantly improving clinical outcomes (7, 8). Furthermore, Aisha Z. Bashir noted that utilizing telemedicine interventions to monitor the recovery of patients with peripheral arterial disease represents a promising and emerging field (9). Currently, however, there is a paucity of established experience and standardized models regarding telemedicine interventions for postoperative rehabilitation in patients with lower extremity arteriosclerosis obliterans. Therefore, this study applied Home-based extended care based on cloud follow-up to the postoperative recovery of lower extremity arteriosclerosis obliterans patients undergoing interventional therapy. We aimed to analyze its impact on disease management, motor function, and quality of life, with the goal of providing a valuable reference for postoperative rehabilitation strategies in this population.

## 2 Study design and methods

### 2.1 Study design

This study employed a prospective cohort study design. A total of 110 patients with lower extremity arteriosclerosis obliterans who underwent interventional treatment in the First Affiliated Hospital of China Medical University from January to December 2023 were selected. According to different postoperative management methods, they were divided into a control group (*n* = 52) and an experimental group (*n* = 58). There were no significant differences in the baseline data between the two groups (*P* > 0.05), as shown in Table 1.

**Table 1:** Comparison of baseline characteristics between two groups.

Variable		Control group (*n* = 52)	Experimental group (*n* = 58)	Statistic	*P* value
Gender	Male	38 (73.08)	42 (72.41)	0.006	0.938
	Female	14 (26.92)	16 (27.59)		
Age		59.36 ± 7.23	58.82 ± 7.51	0.383	0.702
Fontaine stage	Stage I	6 (11.54)	7 (12.07)	−0.013	0.990
	Stage II	13 (25.00)	14 (24.14)		
	Stage III	25 (48.08)	28 (48.28)		
	Stage IV	8 (15.38)	9 (15.52)		
Lesion vessels were	Femoral artery	21 (40.38)	23 (39.66)	−0.102	0.919
	Iliac artery	18 (34.62)	20 (34.48)		
	Popliteal artery and below	13 (25.00)	15 (25.86)		

Inclusion criteria: (1) Met the relevant diagnostic criteria of the “Guidelines for the Treatment of Lower Limb Arteriosclerosis Obliterans.” (2) Confirmed diagnosis of lower extremity arteriosclerosis obliterans via Computed Tomography Angiography. (3) Undergoing interventional surgical treatment for the first time. (4) Possessed clear consciousness and the ability to cooperate with the study protocol, and the patient and their legal guardians signed the informed consent.

Exclusion criteria: (1) Combined with serious liver and kidney diseases. (2) Combined with malignant tumors. (3) Cognitive dysfunction or mental illness.

### 2.2 Routine management intervention

The control group received routine management intervention. Before discharge, patients and their families were given routine discharge guidance, and a postoperative rehabilitation guidance manual designed by medical staff was issued, including medication management, healthy lifestyle establishment, dietary guidance, exercise, complication prevention and self-monitoring, etc. Patients were encouraged to communicate with nursing staff in a timely manner about the problems encountered in postoperative rehabilitation after discharge, and patients were required to have regular follow-up visits. In the first month after discharge, the responsible nurse conducted follow-up supervision by telephone once a week, and then changed to once a month, for 12 consecutive months. The follow-up content included the patient’s condition, medication, diet and exercise, mental health, etc., and timely and targeted solutions were provided for the problems found during the follow-up.

### 2.3 Home-based extended care based on cloud follow-up

The experimental group adopted Home-based extended care based on cloud follow-up.

#### 2.3.1 Established a Home-based extended care team

Establishment of a Home-based Extended Care Team. A multidisciplinary team was established, consisting of two vascular surgeons, one nutritionist, one rehabilitation physician and five nurses. Their respective responsibilities were as follows: the vascular surgeons provided medical treatment guidance and follow-up assessments; the nutritionist was responsible for developing dietary plans and nutritional counseling; the rehabilitation physician oversaw exercise and functional recovery guidance; and the nurses managed post-discharge interventions.

#### 2.3.2 Composition of cloud follow-up

The Home-based extended care team collaboratively developed the cloud-based follow-up platform, which comprises four functional modules: patient files, health education, physician-patient communication, and health monitoring. (1) Patient files: mainly included basic information such as patient name, age, medical history, treatment process and contact information. (2) Health education: Based on the previous clinical practice experience, the team jointly developed the “Guidelines for Postoperative Rehabilitation of Lower Limb Arteriosclerosis Obliterans,” which specifically included knowledge on disease recurrence and postoperative prevention, medication management, dietary guidance, exercise and regular follow-up, and made it into a video lecture for patients and their families to watch and learn. The main topics include: vascular surgeons explained the causes of disease recurrence and the importance of establishing a healthy lifestyle, taking medication on a regular basis and regular follow-up visits. Nutritionist provided guidance on smoking cessation, alcohol restriction, and the adoption of low-salt, low-fat, and low-cholesterol diets, and guided family members to master the method of calculating food exchange portions and prepare meals suitable for the patient’s own conditions, such as patients with diabetes should control total calories and eat a high-fiber diet, and patients with hypertension should adhere to a low-salt, low-fat diet. Rehabilitation physicians personally demonstrated the essentials of Buerger exercise and walking exercise, and explained exercise plans and emergency treatment methods. (3) physician-patient communication: Patients can initiate consultation requests to the Home-based extended care team for medication, diet, exercise, psychological and other issues encountered during postoperative rehabilitation. The Home-based extended care team collected patients’ needs for postoperative rehabilitation, provided rehabilitation guidance and reminded them to have regular follow-up visits. (4) Health monitoring: The patient filled out the “Daily Rehabilitation Log” under the supervision of his family, which mainly includes blood sugar, blood pressure, blood lipids, medication time and dosage, diet and exercise status, etc. The system pre-set reminder rules for blood sugar, blood pressure and blood lipids, and reminded the patient and the Home-based extended care team when the indicators were abnormal.

#### 2.3.3 Implementation of Home-based extended care

(1) Pre-discharge guidance: Before discharge, the nurse guided the patient and his family to master the relevant usage of the cloud follow-up platform and completed the learning of rehabilitation knowledge in the “Health Education” module. (2) Follow-up supervision: In the first month after discharge, the nurse checked the patient’s postoperative rehabilitation implementation status through the “Daily Rehabilitation Log” every day, commented on the medication, diet and exercise records through “doctor-patient communication,” and made appropriate diet and exercise plan adjustments and psychological counseling based on the patient’s feedback information. From the second month after discharge, the nurse changed to weekly summary to check the patient’s postoperative rehabilitation implementation status. Patients with abnormal indicators were required to return for follow-up in time, and vascular surgeons and nurses provided on-site guidance. (3) Special guidance: Combined with the patient’s needs for home rehabilitation such as disease recurrence prevention, exercise and psychological counseling, online video special lectures were organized every month. For example, through the sharing of typical cases of successful postoperative rehabilitation, patients were guided to establish the belief of persisting in postoperative rehabilitation, and patients and their families were encouraged to learn from the experience of typical cases, reduce psychological pressure, and promote the formation of healthy disease management behaviors. (4) Regular follow-up: Vascular surgeons established the follow-up time in the discharge plan. Nurses issued reminders via the platform 3 days prior to each scheduled visit. During these visits, targeted guidance was provided based on the patient’s clinical examination results and disease progression.

### 2.4 Evaluation indicators

#### 2.4.1 Primary outcomes

The primary outcomes included the incidence of vascular restenosis and the Ankle-Brachial Index (ABI). The ABI was measured using a vascular diagnostic device preoperatively and 12 months post-discharge to assess the severity of lower extremity arterial stenosis. Regular color Doppler ultrasound examinations of the lower limbs were performed postoperatively to record the number of restenosis cases within 12 months post-discharge. ABI and lower extremity vascular ultrasonography were performed by technicians who were not involved in this study and remained blinded to the group assignments. Following the acquisition of standard sectional images and blood flow spectra, two independent ultrasound experts (also blinded to the study team) conducted a secondary review of parameters such as stenosis severity and intima-media thickness. Restenosis can be diagnosed when the lumen diameter narrows by more than 50%.

#### 2.4.2 Secondary outcomes

Secondary outcomes encompassed treatment adherence, lower limb motor function, and quality of life.

##### 2.4.2.1 Adherence

Medication adherence: At 6 months post-discharge, the Chinese version of the General Medication Adherence Scale (GMAS) was employed. The scale consists of 11 items, each scored from 0 to 3 on a scale of “always to very good,” with a maximum score of 33. A score <11 indicates poor adherence, while a higher score indicates better adherence (10).

Dietary and exercise adherence: Compliance with dietary control and rehabilitation exercises, as prescribed by the rehabilitation guidelines, was evaluated on a 5-point scale (1–5 points, from “very poor” to “very good”). For dietary adherence, criteria were based on the intake of salt, fats, and smoking status according to clinical guidelines. For exercise adherence, scoring was anchored to the frequency and duration of intermittent walking exercises (e.g., a score of 5 represented ≥5 sessions/week, while a score of 1 indicated no exercise).

Follow-up adherence: The rate of regular follow-up visits within 6 months post-discharge was recorded and compared between groups.

Adherence to medication, dietary control, and rehabilitation exercise was assessed using questionnaires administered by research assistants who were independent of the study team. Furthermore, the Home-based extended care team verified and confirmed the adherence scores of the experimental group by reviewing the Daily Rehabilitation Logs, which documented the patients’ actual execution of postoperative recovery activities. Additionally, adherence to regular follow-up visits was quantified by calculating the ratio of the actual number of completed visits to the total number of scheduled visits.

##### 2.4.2.2 Motor function

Fugl-Meyer assessment (FMA): The FMA (lower extremity section) was used preoperatively and 12 months post-discharge to evaluate motor function. It consists of 7 items and 17 sub-items, each scored from 0 to 2, with a maximum score of 34. Higher scores indicate superior motor function (11).

Six-minute walk distance (6MWD): The 6MWD was conducted to assess exercise tolerance preoperatively and 12 months post-discharge by measuring the maximum distance walked within six minutes (12).

Regarding functional assessments, the FMA and 6MWD were evaluated by rehabilitation therapists who did not participate in the intervention, conducted within an independent assessment room to ensure objectivity.

##### 2.4.2.3 Quality of life

The Vascular Quality of Life Questionnaire (VascuQol) was utilized preoperatively and 12 months post-discharge. The scale comprises 25 items across five domains: pain, symptoms, activities, emotional, and social functions. Each item is scored from 1 to 7. Raw scores are converted into a 7-point scale for each domain, where higher scores represent a better quality of life (13). VascuQol scores were collected via questionnaires administered by research assistants who were independent of the study team.

### 2.5 Statistical analysis

The data of this study were statistically analyzed using SPSS26.0 software. Continuous data following a normal distribution, such as the ABI and quality of life scores, were presented as mean ± standard deviation. Differences between groups were compared using independent samples *t*-tests or ANCOVA adjusted for baseline values. Categorical data, including Fontaine stage and vascular restenosis rates, were expressed as percentages (%) and were analyzed using the rank-sum test or chi-square test, as appropriate. A value of *p* < 0.05 was considered statistically significant.

## 3 Results

### 3.1 Comparison of baseline characteristics

There were no statistically significant differences between the two groups in terms of gender, age, Fontaine stage, or affected vessels (*P*>0.05) (as shown in Table 1).

### 3.2 Comparison of ABI and vascular restenosis

**Figure 1:**
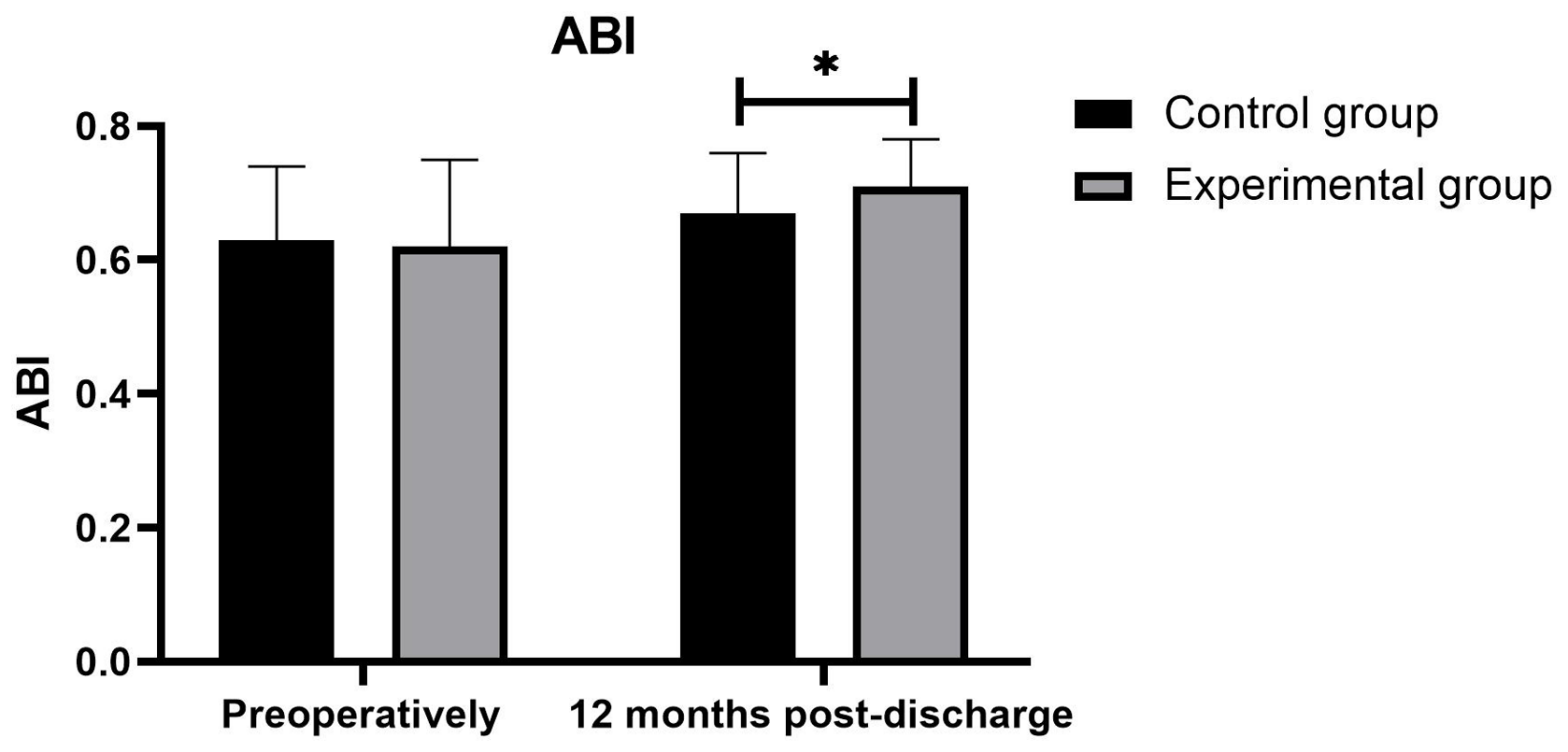
Changes in ABI from baseline to 12 months post-discharge between the two groups. * Indicates *P* < 0.05.

Preoperatively, the ABI showed no significant difference between the two groups (*p* > 0.05). At 12 months post-discharge, the ABI in the experimental group was significantly higher than that of the control group (*p* < 0.05). As shown in Figure 1. After adjusting for baseline values using ANCOVA, the experimental group demonstrated significantly superior brachial-ankle index compared with the control group at 12 months post-discharge (*p* < 0.05). Regarding vascular outcomes, the restenosis rate at 12 months post-discharge was 18.97% in the experimental group, which was significantly lower than the 36.54% observed in the control group (*p* < 0.05) (see Table 2).

**Table 2:** Comparison of ABI and vascular restenosis between two groups.

Variable		Control group (*n* = 52)	Experimental group (*n* = 58)	*t*/χ2	*P* value
ABI	Preoperatively	0.63±0.11	0.62±0.13	−6.219	<0.001
	12 months post-discharge	0.67±0.09	0.71±0.07		
Vascular restenosis		19 (36.54)	11 (18.97)	4.269	0.039

### 3.3 Comparison of adherence

The adherence of the experimental group with medication, diet control, rehabilitation exercise and regular review was higher than that of the control group, and the difference was statistically significant (*p* < 0.05) (as shown in Table 3).

**Table 3:** Comparison of adherence between the two groups.

Variable	Control group (*n* = 52)	Experimental group (*n* = 58)	*t*	*P* value
Medication (points)	28.02±3.29	30.25±2.34	−4.128	<0.001
Dietary control (points)	3.38±0.86	3.91±0.65	−3.669	<0.001
Rehabilitation exercise (points)	3.52±0.91	4.07±0.76	−3.452	0.001
Regular follow-up (%)	94.71±6.34	98.85±2.87	−4.488	<0.001

### 3.4 Comparison of motor function

**Figure 2:**
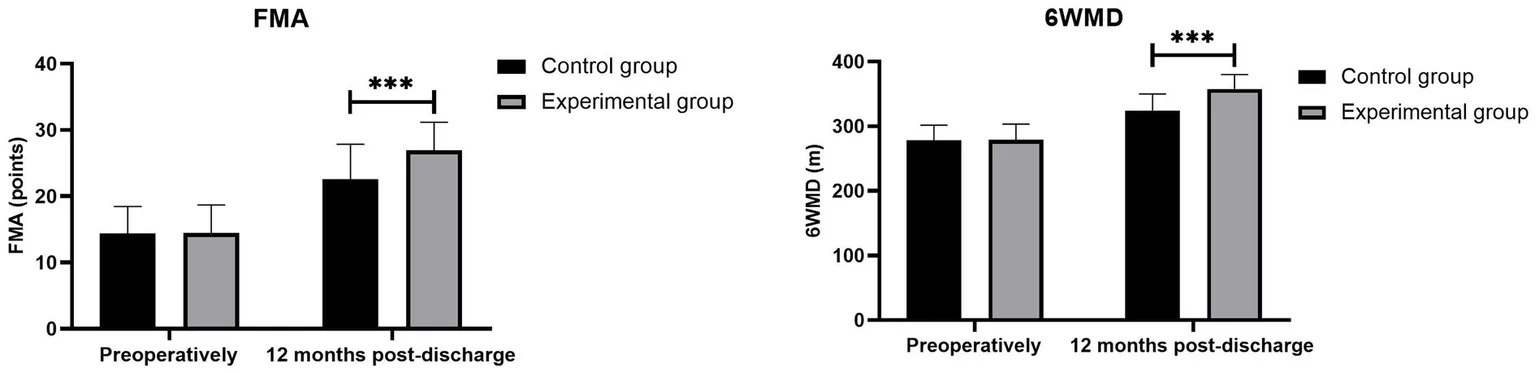
Changes in FMA scores and 6MWD from baseline to 12 months post-discharge between the two groups. *** Indicates *P* < 0.001.

Similarly, no statistically significant differences were observed in FMA scores and 6MWD between the two groups preoperatively (*p* > 0.05). By the 12-month follow-up after discharge, the experimental group consistently outperformed the control group in all the aforementioned indicators (*p* < 0.05). As shown in Figure 2. After adjusting for baseline values using ANCOVA, the experimental group demonstrated significantly superior FMA scores and 6MWD compared with the control group at 12 months post-discharge (*p* < 0.05) (as shown in Table 4).

**Table 4:** Comparison of motor function between the two groups.

Variable		Control group (*n* = 52)	Experimental group (*n* = 58)	*t*	*P* value
FMA (points)	Preoperatively	14.38±4.08	14.45±4.24	−23.925	<0.001
	12 months post-discharge	22.58±5.26	26.95±4.22		
6MWD (m)	Preoperatively	278.01±23.74	279.33±24.09	−137.457	<0.001
	12 months post-discharge	324.56±25.63	357.73±22.44		

### 3.5 Comparison of quality of life scores

**Figure 3:**
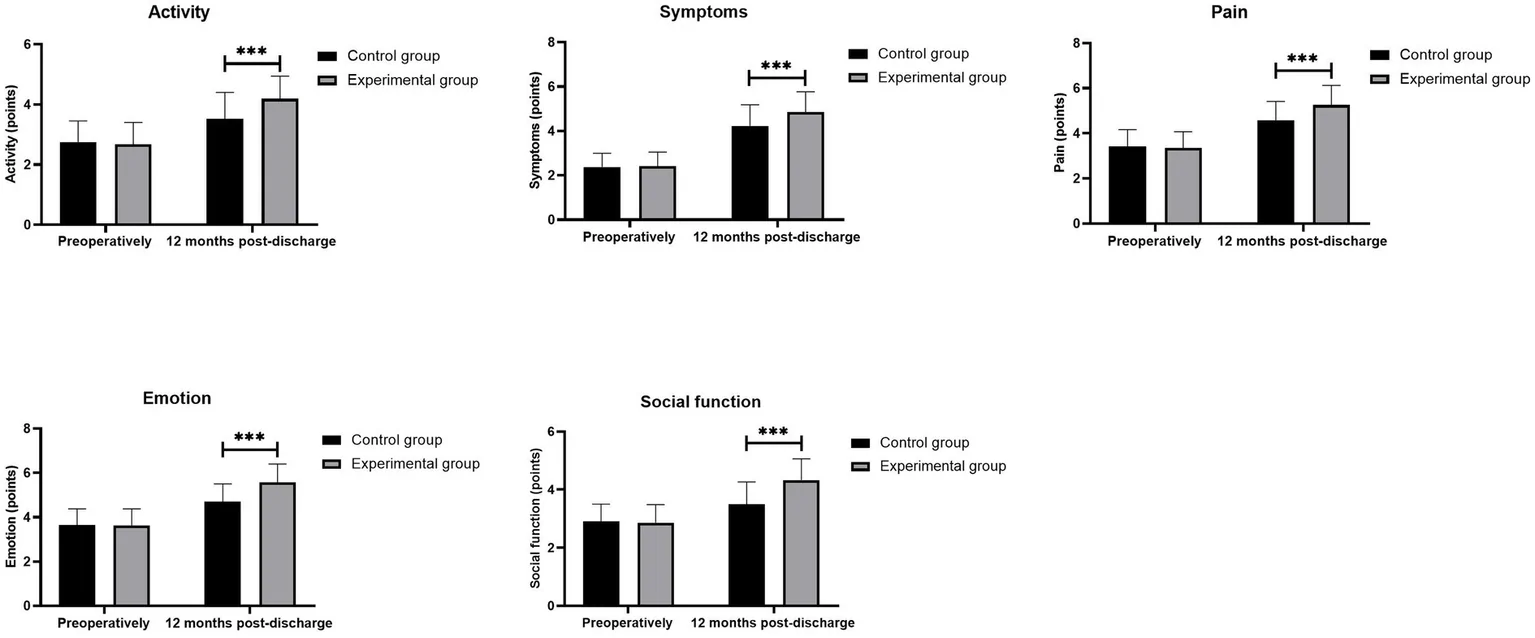
Changes in VascuQol scores from baseline to 12 months post-discharge between the two groups. *** Indicates *P* < 0.001.

Preoperatively, there were no significant differences in all VascuQol subscale scores between the two groups (*p* > 0.05). However, at 12 months post-discharge, the experimental group achieved significantly higher scores across all domains compared to the control group (*p* < 0.05). As shown in Figure 3. After adjusting for baseline values using analysis of ANCOVA, the experimental group demonstrated significantly superior all VascuQol domains compared with the control group at 12 months post-discharge (*p* < 0.05) (see Table 5).

**Table 5:** Comparison of quality of life scores between two groups.

Variable		Control group (*n* = 52)	Experimental group (*n* = 58)	*t*	*P* value
Activity	Preoperatively	2.75±0.70	2.67±0.73	−11.091	<0.001
	12 months post-discharge	3.52±0.88	4.19±0.75		
Symptoms	Preoperatively	2.38±0.62	2.41±0.64	−9.173	<0.001
	12 months post-discharge	4.23±0.95	4.85±0.92		
Pain	Preoperatively	3.42±0.74	3.36±0.71	−13.664	<0.001
	12 months post-discharge	4.58±0.84	5.26±0.86		
Emotion	Preoperatively	3.65±0.73	3.62±0.76	−17.419	<0.001
	12 months post-discharge	4.71±0.79	5.57±0.83		
Social function	Preoperatively	2.90±0.60	2.86±0.63	−13.325	<0.001
	12 months post-discharge	3.50±0.77	4.33±0.73		

## 4 Discussion

Lower extremity arteriosclerosis obliterans is a chronic disease. Lumen restenosis or occlusion may still occur after interventional surgery. Whether the disease recurs is closely related to the control of blood pressure, blood sugar, blood lipids and lifestyle after discharge. Therefore, after discharge, patients should take lipid-lowering and antiplatelet drugs on time and in the right amount, maintain a healthy and scientific lifestyle and have regular follow-up visits (14). If patients cannot effectively implement the discharge plan, the postoperative rehabilitation effect will be affected and a poor prognosis will occur. In this study, the experimental group achieved superior rehabilitation outcomes in terms of restenosis rates and ABI. This improvement can be primarily attributed to the fact that Home-based extended care based on cloud follow-up effectively addresses the limitations imposed by healthcare staff shortages and relative scarcity of medical resources, thereby better meeting patients’ needs for medical services. Under conventional management, patients are typically instructed to follow health manuals for postoperative recovery after discharge. This model is often characterized by low follow-up rates and passive acquisition of knowledge by patients and their families, who lack effective interaction with healthcare providers and struggle to obtain personalized rehabilitation guidance. In contrast, Home-based extended care based on cloud follow-up is a patient-centered, family-involved post-discharge model that represents an optimization and refinement of conventional care. It emphasizes enhanced out-of-hospital guidance and follow-up supervision, improves the practicality, specificity, and professionalism of health education, and advocates for addressing the extended medical service needs of patients.

Patients with lower limb arteriosclerosis obliterans need to adhere to long-term rehabilitation treatment after discharge. As time goes by, most patients will experience problems such as fatigue and burnout, which reduces treatment adherence (15). At 6 months post-discharge, the experimental group demonstrated significantly higher adherence to medication, dietary control, rehabilitation exercise, and regular follow-up visits compared to the control group. This finding is consistent with previous reports on the effectiveness of telemedicine in the rehabilitation management of patients following total knee arthroplasty (16). Home-based extended care based on cloud follow-up delivers health education and specialized guidance through video-based lectures on a dedicated cloud platform. This approach enables patients to intuitively grasp postoperative rehabilitation knowledge, heightens their awareness of out-of-hospital self-management, and encourages the voluntary establishment of a healthy lifestyle. Furthermore, the health monitoring module enhances follow-up supervision, effectively bridging the gap of low follow-up rates in conventional management. By providing comprehensive and continuous oversight, this model facilitates patient adherence to correct daily medication, healthy dietary habits, regular exercise, and scheduled hospital revisits. Additionally, online physician-patient communication allows healthcare providers to address concerns from patients and their families in a timely manner. By providing tailored guidance based on individual needs, this interaction fosters mutual trust and significantly improves treatment adherence.

Relevant studies have indicated that reduced exercise capacity and sluggish peripheral blood flow are critical factors in the pathogenesis of lower extremity arteriosclerosis obliterans (17). Physical exercise can promote the development of collateral circulation in the affected limb, increase local blood perfusion, and correct the state of ischemia and hypoxia. Structured rehabilitation exercise improves motor capacity and effectively mitigates the occurrence of vascular restenosis. Home-based extended care based on cloud follow-up prioritizes the recovery of motor function in the affected limb. Rehabilitation therapists provide personal demonstrations to ensure patients master the essential techniques and methods of exercise, while enhanced follow-up supervision ensures the effective execution of out-of-hospital rehabilitation. At 12 months post-discharge, the FMA scores and 6MWD in the experimental group were superior to those in the control group. These results demonstrate that Home-based extended care based on cloud follow-up achieves superior outcomes in improving postoperative motor function, which was consistent with the view that multimodal web-based interventions play a positive role in enhancing exercise efficacy in patients with cardiopulmonary diseases (18).

Clinical studies have shown that the incidence of restenosis after intervention is relatively high. Patients are prone to anxiety and fear of poor prognosis and disease recurrence after surgery, and have great psychological pressure. At the same time, the onset of the disease has greatly weakened the role of patients in assuming family responsibilities. Especially for families with severe economic pressure, patients find it difficult to obtain support from their family members and will choose to avoid normal social activities. Research on the psychosocial adaptation of patients with lower extremity arteriosclerosis obliterans suggested that psychological assessments and targeted interventions should be strengthened for this population (19). At 12 months post-discharge, the scores for activity, symptoms, pain, emotion, and social function in the VascuQol scale were significantly higher in the experimental group than in the control group. This indicates that a cloud-based home management model provides superior outcomes in promoting psychosocial health and optimizing postoperative quality of life. This improvement can be attributed to the fact that this management model prioritizes mental health construction and recognizes the critical role of family involvement in postoperative recovery. Real-time physician-patient communication combined with family participation allows for an accurate understanding of patients’ internal stressors and needs. By conducting specialized lectures, such as sharing successful rehabilitation case studies, and providing timely, targeted psychological counseling, this approach enhances the cognitive level of both patients and their families. Furthermore, it strengthens patients’ psychological resilience, reduces negative emotions, and fosters a sense of trust in family members and healthcare providers. Consequently, patients can better adapt to role transitions and show improved social functioning. These findings were consistent with evidence suggesting that symptom-management-based rehabilitation interventions can effectively enhance patients’ self-efficacy and quality of life (20).

## 5 Conclusion

Home-based extended care based on cloud follow-up for the rehabilitation of patients with lower extremity arteriosclerosis obliterans can overcome the temporal and spatial constraints of out-of-hospital care. By enhancing the practicality, specificity, and professionalism of rehabilitation guidance, this approach is associated with superior recovery outcomes, including reduced postoperative restenosis, improved lower limb arterial perfusion, and enhanced treatment adherence, motor function, and quality of life.

### 5.1 Limitations

This study is an exploratory research adopting a single-center, non-randomized design with a relatively small sample size. The evaluation of indicators such as medication adherence and quality of life relies on patients’ subjective reports, which may limit the generalizability of the findings. Future efforts should expand the sample size and conduct randomized controlled trials, multicenter validation studies, and cost-effectiveness analyses to further validate the application effectiveness of Home-based extended care based on cloud follow-up in postoperative rehabilitation for lower extremity arterial occlusive disease.

## Data Availability

The raw data supporting the conclusions of this article will be made available by the authors, without undue reservation.
